# Low ventilatory responsiveness to transient hypoxia or breath-holding predicts fast marathon performance in healthy middle-aged and older men

**DOI:** 10.1038/s41598-021-89766-4

**Published:** 2021-05-13

**Authors:** Bartłomiej Paleczny, Rafał Seredyński, Małgorzata Wyciszkiewicz, Adrianna Nowicka-Czudak, Wojciech Łopusiewicz, Dorota Adamiec, Szczepan Wiecha, Dariusz Mroczek, Paweł Chmura, Marek Konefał, Krzysztof Maćkała, Krystyna Chromik, Damian Pawlik, Marcin Andrzejewski, Jan Chmura, Piotr Ponikowski, Beata Ponikowska

**Affiliations:** 1 Department of Physiology, Wroclaw Medical University, Chałubińskiego 10, 50-368 Wrocław, Poland; 2 Department of Physical Education and Health in Biala Podlaska, Education in Warsaw Faculty in Biala Podlaska, Jozef Pilsudski University of Physical, Biala Podlaska, Poland; 3 Department of Human Motor Skills, University School of Physical Education, Wroclaw, Poland; 4 Department of Sport Team Games, University School of Physical Education, Wroclaw, Poland; 5 Department of Track and Field, University School of Physical Education, Wroclaw, Poland; 6 Department of Recreation, University School of Physical Education, Poznan, Poland; 7 Department of Heart Diseases, Wroclaw Medical University, Wroclaw, Poland; 8 Center for Heart Diseases, University Hospital in Wroclaw, Wroclaw, Poland

**Keywords:** Ageing, Neuro-vascular interactions, Autonomic nervous system, Vasodilation

## Abstract

The aim of this study was to test the utility of haemodynamic and autonomic variables (e.g. peripheral chemoreflex sensitivity [PCheS], blood pressure variability [BPV]) for the prediction of individual performance (marathon time and VO_2max_) in older men. The post-competition vasodilation and sympathetic vasomotor tone predict the marathon performance in younger men, but their prognostic relevance in older men remains unknown. The peripheral chemoreflex restrains exercise-induced vasodilation via sympathetically-mediated mechanism, what makes it a plausible candidate for the individual performance marker. 23 men aged ≥ 50 year competing in the Wroclaw Marathon underwent an evaluation of: resting haemodynamic parameters, PCheS with two methods: transient hypoxia and breath-holding test (BHT), cardiac barosensitivity, heart rate variability (HRV) and BPV, plasma renin and aldosterone, VO_2max_ in a cardiopulmonary exercise test (CPET). All tests were conducted twice: before and after the race, except for transient hypoxia and CPET which were performed once, before the race. Fast marathon performance and high VO_2max_ were correlated with: low ventilatory responsiveness to hypoxia (r =  − 0.53, r = 0.67, respectively) and pre-race BHT (r =  − 0.47, r = 0.51, respectively), (1) greater SD of beat-to-beat SBP (all *p* < 0.05). Fast performance was related with an enhanced pre-race vascular response to BHT (r =  − 0.59, *p* = 0.005). The variables found by other studies to predict the marathon performance in younger men: post-competition vasodilation, sympathetic vasomotor tone (LF-BPV) and HRV were not associated with the individual performance in our population. The results suggest that PCheS (ventilatory response) predicts individual performance (marathon time and VO_2max_) in men aged ≥ 50 yeat. Although cause-effect relationship including the role of peripheral chemoreceptors in restraining the post-competition vasodilation via the sympathetic vasoconstrictor outflow may be hypothesized to underline these findings, the lack of correlation between individual performance and both, the post-competition vasodilation and the sympathetic vasomotor tone argues against such explanation. Vascular responsiveness to breath-holding appears to be of certain value for predicting individual performance in this population, however.

## Introduction

Over the past half-century, the marathon has evolved from an Olympic discipline to a mass social phenomenon, with thousands of participants competing in very popular runs organized in nearly every city throughout the world today^1,2^. The tremendous growth in popularity of the marathon running can be illustrated by the number of finishers in the New York City Marathon, the world’s largest one: > 12 K in 1980, > 30 K in 1997, > 44 K in 2010 and > 53 K in 2019^3^. As another example, nearly half a million runners have submitted their application for the 2020 London Marathon^4^. As a consequence of the boom, researches on physiology and training aspects of marathon running (in particular, fast performance determinants) have been attracting increasing attention of thousands of professional as well as non-professional athletes around the world^1,5^.

Noteworthy, middle-aged and older adults represent a substantial and growing proportion of the marathon-running population. Over the years, the mean age of the finishers of the New York City Marathon increased from ~ 35 y in the 1970s to ~ 41 y in the 2010s^6^ with almost 30% of all male finishers in 2019 being 50 years old or older^7^. This underscores the need for further in-depth studies on the physiology of marathon running with advanced age.

Given that the autonomic nervous system (ANS) is a master regulator of visceral functions at rest and during the exercise, an indirect “autonomic monitoring” (including ANS surrogate measures: haemodynamic parameters and heart and blood pressure variability analysis) has been proposed as a useful tool for predicting the performance^1,5^. In an elegant field study by Gratze et al.^8^ five haemodynamic monitors were used simultaneously to assess circulatory and ANS parameters in a group of 51 male marathon runners within 2 h after completion of the Graz marathon. The authors reported enhanced postcompetition vasodilation and low sympathetic vasomotor tone to be major predictors of fast marathon performance. However, subjects studied by Gratze et al. were relatively young (mean age: ~ 40 y, range: 26–57 y). Given that aging is characterized by an impaired endothelium-dependent vasodilation^9^ and sympathetic neurovascular transduction^10,11^ and by an increased basal sympathetic drive^12,13^, these results do not necessarily apply to men over 50 years of age.

Secondly, most previous studies in this field have assessed cardiac baroreflex sensitivity as a plausible marker of athletic performance^8,14^, but have ignored the peripheral chemoreceptor mechanism, possibly due to technical difficulties. However, the observations that the peripheral chemoreflex restrain the exercise-induced vasodilation via increased sympathetic vasoconstrictor outflow^15–17^ provide strong rationale for considering peripheral chemoreflex sensitivity (PCheS) as a possible important predictor of marathon performance. Thirdly, plasma renin activity was shown to be inversely associated with subject’s physical fitness^18,19^ and was proposed as a marker of sympathetic activity^20^, given its putative role in the autonomic regulation of cardiovascular control^21^. Therefore, the components of renin–angiotensin–aldosterone system were included in the study as potential candidates for the predictors of individual performance.

To address these knowledge gaps, we used an approach similar to that described by Gratze et al.^8^ in a group of men aged 50 years and older competing in the 37th PKO Wroclaw Marathon (Wroclaw, Poland, 19th September 2019). We aimed to test whether the parameters of individual performance (the competition time and maximal oxygen consumption, VO_2max_, as assessed with the treadmill cardiopulmonary test) in healthy middle-aged and older men are associated with: (1) haemodynamic parameters measured in a continuous and non-invasive fashion, (2) spontaneous cardiac baroreflex sensitivity (cBRS) estimated non-invasively with the sequence method, (3) PCheS as assessed by the ‘gold standard’ transient hypoxia method and recently proposed breath-holding method, (4) autonomic balance as assessed by heart rate variability (HRV) and blood pressure variability (BPV) analysis and (5) plasma renin and aldosterone levels. All the tests listed were conducted twice: before and immediately after the race, except for the transient hypoxia test and the cardiopulmonary exercise test which were performed once, before the race.

## Methods

### Ethical approval and study population

Twenty-six healthy men aged 50 years and older participating in the 37th PKO Wroclaw Marathon (Wroclaw, Poland, 19th September 2019) were recruited prospectively. All participants were free from any acute illness or chronic disease and not taking regular medication. The study protocol was approved by the local Institutional Ethics Committee (Senacka Komisja ds. Badań Naukowych przy Akademii Wychowania Fizycznego we Wrocławiu). All participants gave written informed consent. The research was conducted in accordance with the Declaration of Helsinki.

Out of 26 men enrolled, one subject failed to complete the marathon and the post-race haemodynamic recordings collected from two other subjects were of unacceptable quality due to technical issues (frequent Nexfin finger-cuff oscillations). These subjects were excluded from the analysis and the final sample included 23 participants. For two subjects, oscillations of the Nexfin finger cuff were limited to the post-race BHT. Therefore, we decided to exclude these subjects from the analysis of BHT-SVR slope only, given that none of the other parameters analysed in the study were affected. Blood sample cannot be obtained from one patient after the race.

Given that the study protocol required all the laboratory experiments to be carried out on the same day (4 days before the run) and that the transient hypoxia test is a time-consuming procedure, the test was conducted in a subgroup of the study participants (N = 18).

### Study protocol

**Figure 1:**
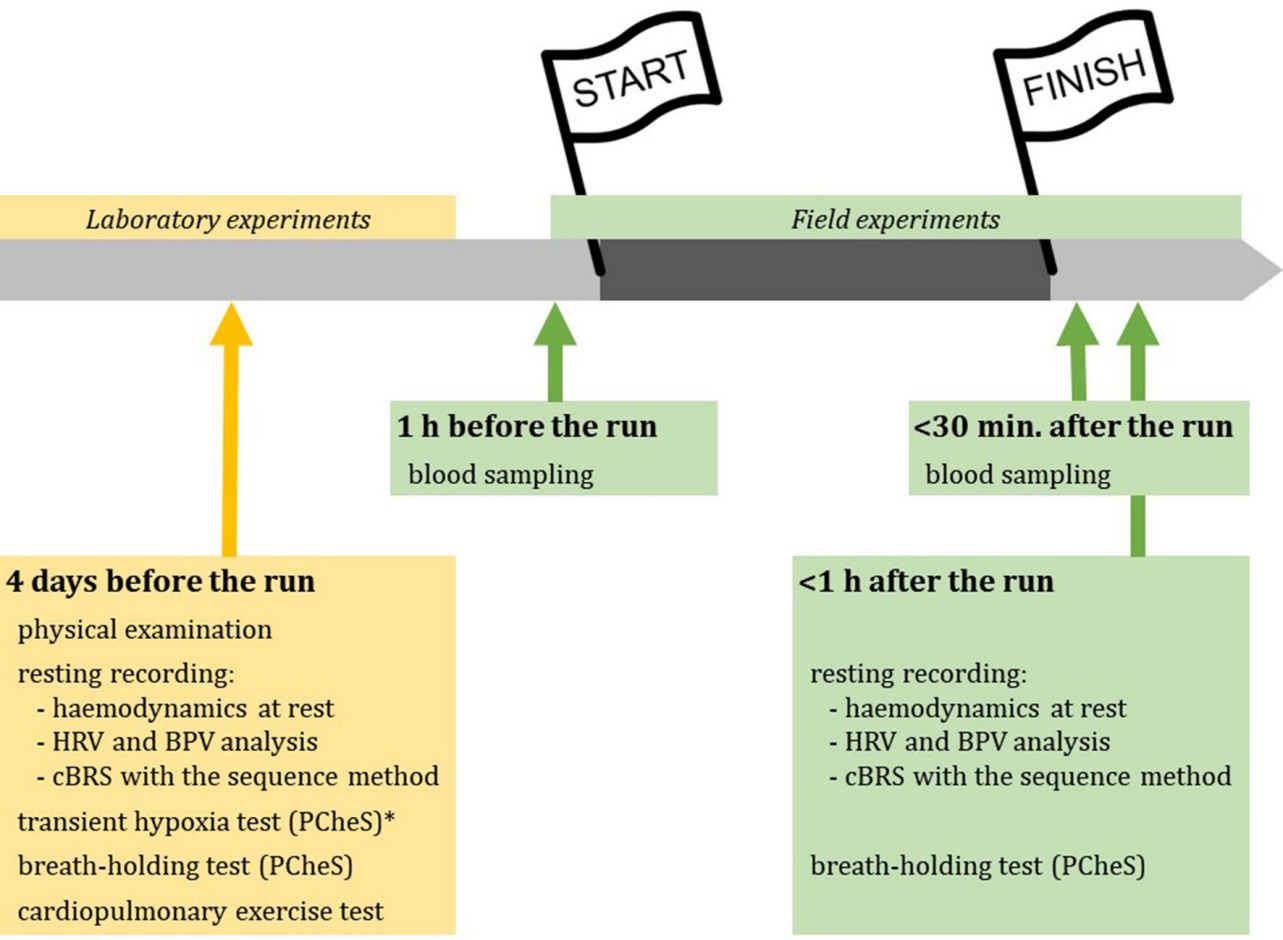
Schematic representation of the study protocol.

Figure 1 outlines the study protocol. All laboratory and field experiments were performed in the order listed below.

#### Laboratory experiments

All participants were admitted to the laboratory (Department of Physiology, Wroclaw Medical University) once, 4 days before the marathon, for: standard physical examination, measurement of haemodynamic parameters at rest (*resting recording*), the assessment of PCheS with the transient hypoxia method and/or the breath-holding method and the cardiopulmonary exercise testing on a treadmill.

#### Field experiments

On the day of the marathon, venous blood sample was collected by venepuncture twice, within 1 h before the race and within 30 min after the race. Blood samples were centrifuged immediately and the plasma separated and frozen at − 70 °C for subsequent analysis. The measurement of haemodynamic parameters at rest (*resting recording*) and the assessment of PCheS with the breath-holding method were performed once, within 1 h after the race.

Given that the transient hypoxia test is time-consuming (one experimental session lasts typically 40–60 min) and technically demanding (e.g. requires breathing circuit and nitrogen tank), the field assessment of PCheS after the race was relied on the BHT only.

*Resting recordings* before and after the marathon were used for the evaluation of: (1) cBRS with the sequence method, and (2) HRV and BPV analysis. Five-minute segment of the entire > 10-min resting recording, free of movement artefacts, was selected and used for the calculation of cBRS, HRV and BPV.

### Measurement of haemodynamic parameters at rest

*Beat-to-beat cardiovascular parameters* heart rate (HR, beats/min.), RR interval (RRI, ms), systolic (SBP, mmHg), diastolic (DBP, mmHg) and mean arterial blood pressure (MAP, mmHg), stroke volume (SV, mL/beat), cardiac output (CO, L/min), and systemic vascular resistance (SVR, dyn × s/cm^5^) were derived from the Nexfin monitor (BMEYE B.V., The Netherlands). Nexfin employs the volume-clamp technique to measure finger arterial blood pressure in a beat-to-beat fashion^22^ and built-in algorithms to reconstruct brachial pressure from finger pressure waveform^23^. The height correction unit of the Nexfin device is used to compensate for a difference in height (and thereby, hydrostatic pressure) between the finger and the heart. SV, CO and SVR are estimated employing the Nexfin pulse-contour method^23^. Nexfin HR measurement was based on finger pressure waveform analysis.

10-min *resting recording* of haemodynamic parameters was performed with the subject lying supine. All haemodynamic parameters were monitored for > 3 min prior to the *resting recording* to ensure stabilization of haemodynamics and data from this period were not included in the analysis.

### Baroreflex sensitivity assessment using the sequence method

Beat-to-beat SBP and RRI from the *resting recording* were used for the cBRS estimation by the sequence method^24^. All sequences of ≥ 3 cardiac cycles in which SBP increases or decreases by ≥ 1 mmHg were accompanied by, respectively, RRI lengthening or shortening by ≥ 5 ms, were isolated and slope of the regression line relating SBP and RRI was computed for each sequence isolated. SBP values were paired with accompanying RRI values (SBP–ECG delay of 0 beat), as this approach was reported to be the most appropriate^25^. Sequences with R^2^ < 0.85 were excluded from the analysis. cBRS (ms/mmHg) was computed as the average of all slopes.

### Peripheral chemoreflex sensitivity assessment

The transient hypoxia test^26,27^ is a well-established method for the evaluation of PCheS, that is believed to represent a relatively “pure” response from peripheral chemoreceptors, unlike the steady-state hypoxia tests reflecting a more systemic response to hypoxia^28^. However, due to technical difficulties, it cannot be performed in the field. Therefore, a novel promising, yet not fully established method, the breath-holding test^29,30^ has been used to assess the PCheS before and after the race, in addition to the transient hypoxia testing carried out before the *race* only.

### Peripheral chemoreflex sensitivity assessment using the transient hypoxia method

The transient hypoxia method was used for the evaluation of PCheS, as described previously^26,27^. The subject lies supine and breaths through the oronasal mask (7450 series V2 oro-nasal mask, Hans Rudolph Inc., USA) connected to a two-way non-rebreathing valve (Hans Rudolph Inc.). The inspiratory side of the valve is connected to a three-way stopcock-type valve (Hans Rudolph Inc.), allowing the operator to switch between room air and 60-L bag filled with pure nitrogen.

All haemodynamic parameters were measured with the Nexfin monitor as described above (see: Measurement of haemodynamic parameters at rest), except for HR, which was calculated from the ECG (BioAmp, ADInstruments, New Zealand). Respiratory flow was measured continuously with a 1000 L/min flowhead (MLT3000L, ADInstruments) placed on the inspiratory side of the breathing circuit and a differential pressure transducer (FE141 Spirometer, ADInstruments), and used to calculate instantaneous values of tidal volume (V_T_, L), breathing rate (BR, breaths/min) and minute ventilation (V_Ins_, L/min). Blood oxygen saturation (SpO_2_, %) was measured with the Nexfin monitor and an ear probe (Masimo Corporation, USA). All data were recorded at a sampling frequency of 1 kHz using data acquisition hardware (PowerLab 16/30, ADInstruments) and software (LabChart 8, ADInstruments).

During the test, the subject is repeatedly switched from breathing room air to pure N_2_ from the bag and then back to room air, in order to evoke a transient decrease in SpO_2_. The test includes four to seven N_2_-breathing episodes, each lasting three to eight breaths. The length of the consecutive N_2_-breathing episodes is changed randomly in order to collect a range of SpO_2_ decreases (SpO_2_ nadirs) from 70 to 90%.

### Quantifying respiratory responses to transient hypoxia

The arithmetic average of three largest consecutive V_Ins_ values (referring to three consecutive breaths) within a time-window starting 5 s before the SpO_2_ nadir and ending 30 s after the SpO_2_ nadir was calculated for each N_2_-breathing episode and plotted against corresponding SpO_2_ nadir (*post-hypoxic* V_Ins_-to-SpO_2_). *Pre-hypoxic* V_Ins_-to-SpO_2_ was calculated from 90-s period immediately preceding N_2_-breathing episode, by plotting 90-s average of V_Ins_ against 90-s average of SpO_2_. Finally, a regression line relating all *pre-* and *post-hypoxic* V_Ins_-to-SpO_2_ was drawn and the slope of the line was defined as the minute ventilation response to transient hypoxia (V_Ins_-Hypo, L/min/SpO_2_%). The tidal volume response to transient hypoxia (V_T_-Hypo, L/SpO_2_%) was calculated analogously, but VT was used instead of V_Ins_. Given the inverse relation between SpO_2_ and breathing (V_Ins_ and VT), lower (more negative) values of the slope (V_Ins_-Hypo and V_T_-Hypo) indicate greater ventilatory responsiveness to hypoxia, whereas higher (less negative) values indicate lower ventilatory responsiveness to hypoxia.

### Quantifying haemodynamic responses to transient hypoxia

To reduce the possible confounding effect of spontaneous (e.g. respiratory-induced) fluctuations in haemodynamic parameters, a moving average (10-s window) was used to smooth the data^28^. The following haemodynamic responses to transient hypoxia: heart rate response (HR-Hypo, beats/min/SpO_2_%), systolic blood pressure response (SBP-Hypo, mmHg/SpO_2_%), mean arterial blood pressure response (MAP-Hypo, mmHg/SpO_2_%), and systemic vascular resistance response (SVR-Hypo, dyn × s/cm^5^/SpO_2_%) were calculated analogously to the minute ventilation to transient hypoxia, using HR, SBP, MAP or SVR, respectively, instead of V_Ins_. The only difference was that the single highest value (and not the average of three consecutive largest values) of HR, SBP or MAP within the time-window was used for calculation of HR-Hypo, SBP-Hypo and MAP-Hypo, respectively, and the single lowest value of SVR within the time-window was used for calculation of SVR-Hypo.

### Peripheral chemoreflex sensitivity assessment using the breath-holding method

The breath-holding test (BHT) for PCheS evaluation, as proposed by Trembach and Zabolotskikh^29^, was performed either immediately after the resting recording (in subjects not undergoing PCheS assessment with the transient hypoxia test) or immediately after PCheS assessment with the transient hypoxia. In both scenarios, the measuring instruments used in the preceding stage were utilized, except for the oronasal mask which was removed after completion of the transient hypoxia test.

The test was performed with the subject lying supine. The subject was asked to hold his/her breath at about 2/3 of the vital capacity for as long as possible, and the duration of the voluntary apnoea was measured from the end of inspiration to the onset of reflex contractions of the diaphragm as assessed by palpation. The breath-hold manoeuvre was performed two to three times with ≥ 5 min pause between consecutive runs and the average duration of the voluntary apnoea was taken as a measure of PCheS (BHT apnoea duration, s). Several papers reported high agreement between BHT and single-breath CO_2_ method for PCheS evaluation in healthy subjects^29,31^ and heart failure patients^30^, however to the authors’ knowledge, the agreement between BHT and the transient hypoxia test has not yet been investigated.

**Figure 2:**
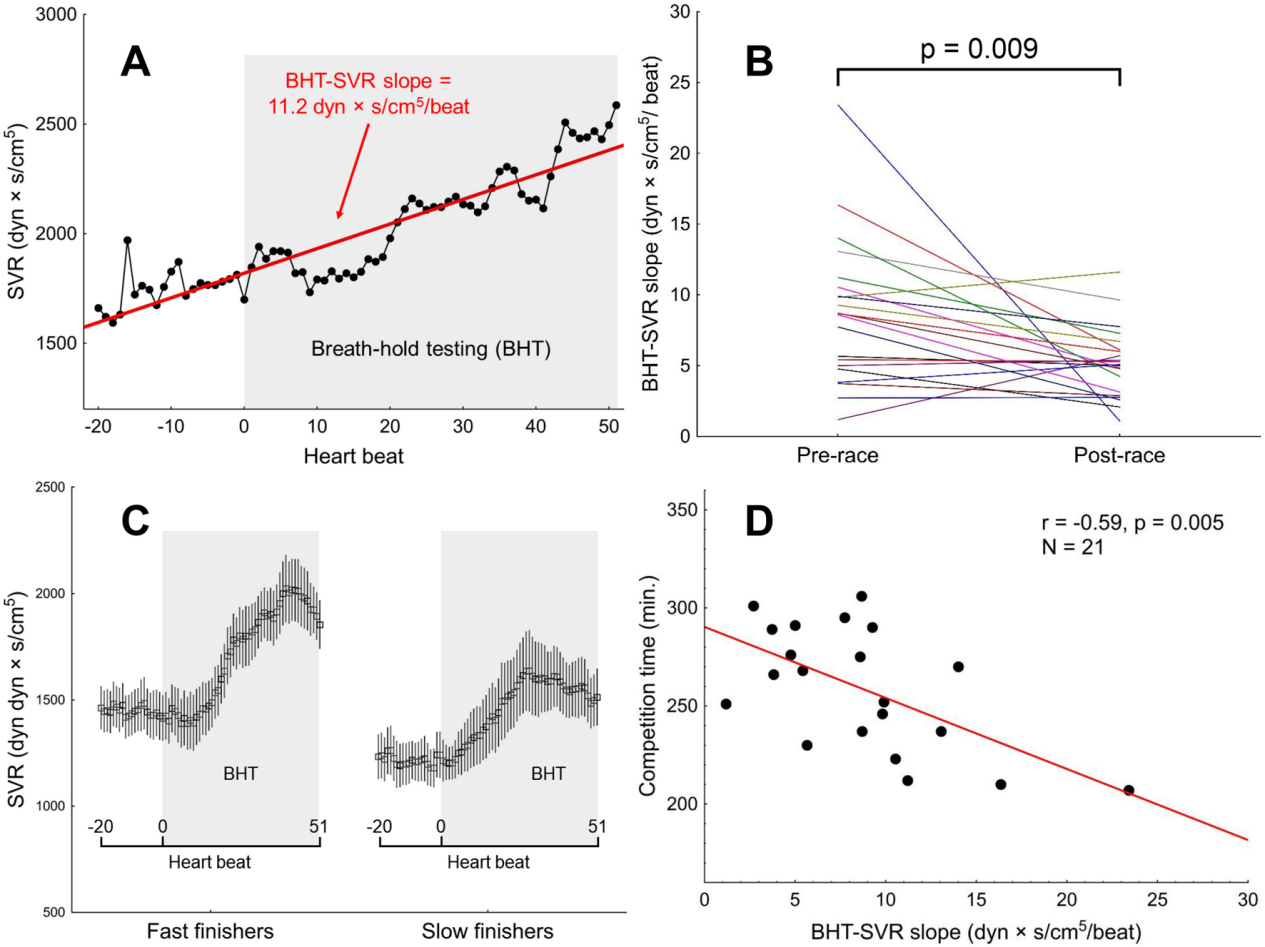
Vascular (SVR) responsiveness to the breath-holding test (BHT). (**A**) Calculation of the SVR response to a single voluntary apnoea episode. (**B**) Pre- and post-race values of the BHT-SVR slope. (**C**) Beat-to-beat SVR response to the BHT in fast finishers versus slow finishers (classified according to the median of the marathon time; whiskers indicate standard error). (**D**) The competition time plotted against the pre-race BHT-SVR slope.

Although not considered in the original version of the BHT, SVR response to the breath-holding was calculated from beat-to-beat recordings, covering the interval between 20th heart beat before the onset of the apnoea and 51st heart beat following the onset of the apnoea (72 beats in total), and expressed as the slope of the regression line. The procedure is shown on Fig. 2A. Responsiveness to BHT in terms of SVR (BHT-SVR slope, dyn × s/cm^5^/ beat) was defined as a mean of all slopes calculated from all episodes of voluntary apnoea.

### Heart rate variability and blood pressure variability analysis

Ectopic beats, if present, were corrected by interpolation. Standard autoregressive methods were used for the spectral analysis of HRV^32^. The following HRV and BPV indices were computed: standard deviation of RRIs (SDNN, ms), the percentage of adjacent RRIs with a difference of duration > 50 ms (pNN50, %), the power of HRV spectrum within low-frequency range (0.04–0.15 Hz) and high-frequency range (0.15–0.40 Hz) expressed in normalized units (LF-HRV and HF-HRV, respectively, nu), standard deviation of beat-to-beat SBP (BPV-SD, mmHg), the power of DBP variability spectrum within low-frequency range (0.05–0.17 Hz, LF-BPV)^14,32^. HRV parameters were calculated based on Nexfin-derived blood pressure signal. SDNN is believed to reflect overall autonomic regulation of the heart function; pNN50 and HF-HRV are markers of the vagal tone, while the physiological background of LF-HRV includes sympathetic or mixed sympathetic and parasympathetic activity^33^.

### Maximal oxygen consumption measurement

All subjects underwent a maximal cardiopulmonary exercise test on a motorised treadmill (Trackmaster TMX425, Full Vision, Inc., KS, USA). After a 5-min running at 8 km/h (warm-up), the protocol started and a treadmill speed was increased by 1 km/h every 2 min, in a stepwise fashion. The treadmill inclination was kept constant at 1°. The protocol was continued until exhaustion.

Oxygen uptake (VO_2_, mL/min/kg), and instantaneous minute ventilation (VE, L/min) were measured breath-by-breath (Cosmed Quark CPET, Rome, Italy) and averaged every 30 s. The highest values of VO_2_ and VE were taken as VO_2max_ (mL/kg/min) and maximal minute ventilation (VE_max_, mL/min/kg) respectively.

### Blood chemistry

Plasma renin (µIU/mL) and aldosterone (ng/dL) levels were measured using chemiluminescence immunoassay (CLIA) technique on an automated analyser (Liaison, Diasorin, Dartford, UK).

### Data and statistical analyses

Statistica (Statsoft, USA), Matlab 2016a (Mathworks, USA), LabChart (ADInstruments) and CardioSeries Software (http://www.danielpenteado.com) were used for data analysis. Kolmogorov–Smirnov test was used to assess the normality of distribution of continuous variables and the results were ensured by visual inspection of frequency histograms. For the parameters measured twice, before and after the competition, pre-race values were tested only. Data are presented as mean ± standard deviation (for continuous normally distributed variables), median with lower and upper quartile (for continuous variables that did not follow a normal distribution) or number and percentage (for categorical data). For the parameters measured twice (before and after the race), mean percentage difference ± standard deviation was presented.

Paired Student’s *t* test was used for within-subject comparisons. Pearson’s linear correlation coefficient was used to assess the relations between variables, in particular between the parameters of individual performance (the marathon time and VO_2max_) and the haemodynamic and autonomic parameters (predictors). In order to reduce the number of correlations carried out and avoid redundancy a priori, we decided to analyse the correlations for: (1) pre-race values of all haemodynamic and autonomic parameters, (2) pre- versus post-race changes for these parameters only which had changed significantly after the race. Therefore, neither post-race values of all parameters nor the pre- versus post-race changes of the parameters not affected by the competition were included in the correlation analysis.

For all haemodynamic and autonomic parameters, a general linear model was used to explore the effect of interaction between pre-race value of a given parameter and pre- versus post-race change in the given parameter ([pre-race × change] interaction) on the dependent variable: the marathon time or VO_2max_. To further investigate the nature of the observed significant pre-race × change interactions for a given parameter, the subjects were dichotomized according to the median of the pre-race value of the parameter, and linear correlations between the pre- versus post-race change versus the marathon time or VO_2max_ were assessed in both groups.

Due to the exploratory nature of the study, we decided not to adjust the significance level to account for multiple testing. A value of *p* < 0.05 was considered statistically significant.

In addition to the analysis of correlation, we attempted to compare the marathon time between the subjects with PCheS within the normal range (normal PCheS) and the subjects with peripheral chemoreceptor hypersensitivity (high PCheS), according to the cut-off value of V_Ins_-Hypo commonly used in clinical studies on PCheS in heart failure patients^34–36^. Based on these studies, the value of V_Ins_-Hypo of − 0.7 L/min/SpO_2_% was used to allocate the subjects into the normal PCheS group (V_Ins_-Hypo between zero and − 0.7 L/min/SpO_2_%) or high PCheS group (V_Ins_-Hypo more negative than − 0.7 L/min/SpO_2_%).

Last, in order to better visualize the SVR response to breath-holding in slow versus fast finishers, the subjects were dichotomized according to the median of the marathon time in our population (266 min.).

### Ethics approval

The study protocol was approved by the local Institutional Ethics Committee (Senacka Komisja ds. Badań Naukowych przy Akademii Wychowania Fizycznego we Wrocławiu). The research was conducted in accordance with the Declaration of Helsinki.

### Consent to participate

All participants gave written informed consent.

### Consent for publication

All participants gave written informed consent.

## Results

Baseline characteristics of the study participants are summarized in Table 1.

**Table 1:** Baseline characteristics of the examined men.

	N	
**Age, years**	23	56[52; 63]
50–59 years	15	
60–69 years	6	
70–74 years	2	
Body mass index, kg/m^2^	23	24.1 ± 2.1
Personal best time in marathon, min	23	233 ± 43
Marathon time, min	23	256 ± 34
Duration of training, years	23	9 ± 5
Weekly running distance, km	23	51 ± 24
VO_2max_, mL/min/kg	23	44.0 ± 3.8
VE_max_, L/min	23	129 ± 20

Data are presented as mean ± SD or median with lower and upper quartile where appropriate;

VO_2max_, maximal oxygen consumption as assessed with cardiopulmonary exercise test; VE_max_, maximal minute ventilation during the cardiopulmonary exercise test.

### Haemodynamic parameters

As expected, the post-race CO was higher, and the post-race values of RRI, SBP, MAP, SV and SVR were lower as compared with the pre-race values (all *p* < 0.01, Table 2).

**Table 2:** Pre- and post-race values of haemodynamic parameters in the examined men (N = 23).

	Pre-race	Post-race	% Change	P value
RRI, ms	1064 ± 163	783 ± 99	− 25 ± 10	< 0.001
SBP, mmHg	133 ± 20	118 ± 16	− 10 ± 17	< 0.01
DBP, mmHg	70 ± 11	66 ± 8	− 3 ± 16	0.14
MAP, mmHg	94 ± 14	82 ± 1	− 10 ± 14	< 0.001
SV, mL/beat	97 ± 11	85 ± 11	− 11 ± 11	< 0.001
CO, L/min	5.6 ± 1.0	6.6 ± 1.2	21 ± 25	< 0.001
SVR, dyn × s/cm^5^	1383 ± 301	1046 ± 200	− 22 ± 20	< 0.001

Data are presented as mean ± SD;

RRI, RR interval; SBP, systolic blood pressure; DBP, diastolic blood pressure; MAP, mean arterial blood pressure; SV, stroke volume; CO, cardiac output; SVR, systemic vascular resistance.

Pre- versus post-race change in RRI was correlated with both, the competition time (r = 0.46, *p* = 0.02) and VO_2max_ (r =  − 0.46, *p* = 0.03), while the correlation between the change in CO and the competition time was not statistically significant (r =  − 0.40, *p* = 0.06). No other relations between the haemodynamic parameters and the parameters of individual performance (the competition time or VO_2max_) were found (all *p* ≥ 0.15, Table 3). Furthermore, no significant pre-race × change interactions were identified (Table 3).

**Table 3:** Correlations between the parameters of individual performance (the competition time and VO_2max_) and the haemodynamic variables (pre-race values and pre-vs. post-race changes).

	Competition time			VO_2max_		
	Pre-race	% Change	Pre-race × change interaction	Pre-race	% Change	Pre-race × change interaction
RRI	− 0.21 *p* = 0.33	0.46 *p* = 0.02	*p* = 0.51	0.24 *p* = 0.28	− 0.46 *p* = 0.03	*p* = 0.47
SBP	− 0.12 *p* = 0.59	− 0.12 *p* = 0.57	*p* = 0.07	− 0.05 *p* = 0.83	0.12 *p* = 0.59	*p* = 0.10
MAP	− 0.11 *p* = 0.62	0.11 *p* = 0.63	*p* = 0.66	− 0.11 *p* = 0.60	0.15 *p* = 0.51	*p* = 0.71
SV	0.06 *p* = 0.77	− 0.10 *p* = 0.65	*p* = 0.66	0.31 *p* = 0.15	− 0.12 *p* = 0.58	*p* = 0.98
CO	0.19 *p* = 0.39	− 0.40 *p* = 0.06	*p* = 0.08	0.03 *p* = 0.89	0.21 *p* = 0.33	*p* = 0.26
SVR	− 0.26 *p* = 0.23	0.26 *p* = 0.23	*p* = 0.79	− 0.06 *p* = 0.80	− 0.05 *p* = 0.83	*p* = 0.20

*r* Pearson’s linear correlation coefficients and *p* values are presented for correlations with pre-race values and % change; *p* values for general linear model are presented for pre-race × change interaction.

RRI, RR interval; SBP, systolic blood pressure; DBP, diastolic blood pressure; MAP, mean arterial blood pressure; SV, stroke volume; CO, cardiac output; SVR, systemic vascular resistance.

**Figure 3:**
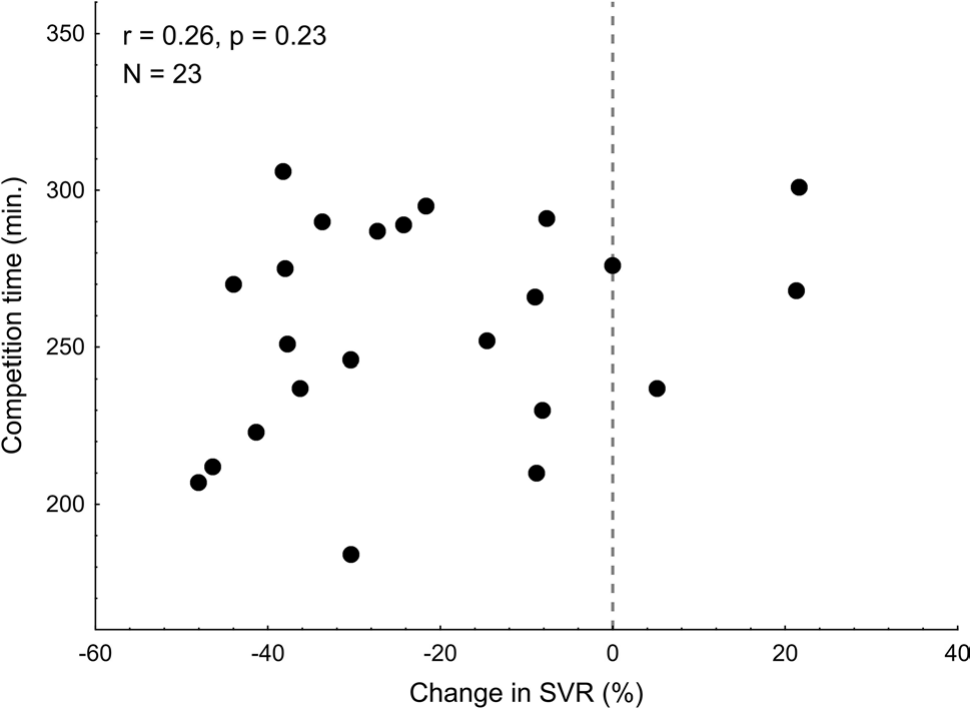
The competition time plotted against the pre- versus post-race change in SVR.

Given that the post-exercise alterations in total peripheral resistance have been shown previously to be associated with marathon performance^8^, scatter plot of pre- versus post-race change in SVR against competition time was presented (Fig. 3).

### Spontaneous cardiac baroreflex sensitivity

cBRS decreased after the competition (Table 4). Neither pre-race cBRS nor the change in cBRS were correlated to the competition time (r =  − 0.18, *p* = 0.42 and r = 0.06, *p* = 0.79, respectively) or VO_2max_ (r = 0.01, *p* = 0.96 and r =  − 0.30, *p* = 0.17, respectively). Furthermore, no significant pre-race × change interactions were found (*p* = 0.23 and 0.84 for the competition time and VO_2max_, respectively).

**Table 4:** Pre- and post-race values of autonomic parameters in the examined men.

	N				
**Spontaneous baroreflex sensitivity**					
cBRS, ms/mmHg	23	12.0 ± 6.0	6.1 ± 2.5	− 39 ± 40	< 0.001
**Peripheral chemoreflex sensitivity by the transient hypoxia test**					
V_Ins_-Hypo, L/min/SpO_2_%	18	− 0.42 ± 0.32			
V_T_-Hypo, L/SpO_2_%	18	− 0.037 ± 0.029			
HR-Hypo, beats/min/SpO_2_%	18	− 0.54 ± 0.16			
SBP-Hypo, mmHg/SpO_2_%	18	− 0.94 ± 0.43			
MAP-Hypo, mmHg/SpO_2_%	18	− 0.67 ± 0.29			
SVR-Hypo, dyn × s/cm^5^/SpO_2_%	18	14.6 ± 5.3			
**Peripheral chemoreflex sensitivity by the breath-holding test**					
BHT apnoea time, s	23	58 ± 20	54 ± 14	1 ± 38	0.27
BHT-SVR slope, dyn × s/cm^5^/beat	21	8.7 ± 5.1	5.2 ± 2.5	− 8 ± 95	< 0.01
**Heart rate variability**					
SDNN, ms	23	52 ± 17	27 ± 11	− 40 ± 38	< 0.001
pNN50, %	23	16 ± 17	1 ± 2	− 86 ± 21	< 0.001
LF-HRV, nu	23	62 ± 17	82 ± 13	41 ± 42	< 0.001
HF-HRV, nu	23	38 ± 17	18 ± 13	− 50 ± 30	< 0.001
**Blood pressure variability**					
BPV-SD, mmHg	23	5.8 ± 1.7	5.2 ± 1.6	− 5 ± 43	0.11
LF-BPV, %	23	41 ± 12	57 ± 14	48 ± 48	< 0.001
**Blood chemistry**					
Renin, µIU/mL	22	16 ± 9	162 ± 75	1071 ± 690	< 0.001
Aldosterone, ng/dL	22	7 ± 2	37 ± 16	462 ± 292	< 0.001

Data are presented as mean ± SD;

cBRS, spontaneous cardiac baroreflex sensitivity as assessed with the sequence method; V_Ins_-Hypo, minute ventilation response to transient hypoxia; V_T_-Hypo, tidal volume response to transient hypoxia; HR-Hypo, heart rate response to transient hypoxia; SBP-Hypo, systolic blood pressure response to transient hypoxia; MAP-Hypo, mean arterial blood pressure response to transient hypoxia; SVR-Hypo, systemic vascular resistance response to transient hypoxia; BHT apnoea time, duration of the voluntary apnoea in the breath-holding test; BHT-SVR slope, SVR responsiveness in the breath-holding test; SDNN, standard deviation of RRIs; pNN50, the percentage of adjacent RRIs with a difference of duration > 50 ms; LF-HRV, the power of HRV spectrum within low-frequency range; HF-HRV, the power of HRV spectrum within high-frequency range; BPV-SD, standard deviation of beat-to-beat SBP; LF-BPV, the power of DBP variability spectrum within low-frequency range.

### Peripheral chemoreflex sensitivity

The pre-race values for PCheS as assessed with the transient hypoxia test and the pre- and post-race values for PCheS as assessed with the BHT were presented in Table 4. BHT apnoea time did not change after the race, while the BHT-SVR slope was lowered (Table 4, Fig. 2B).

**Figure 4:**
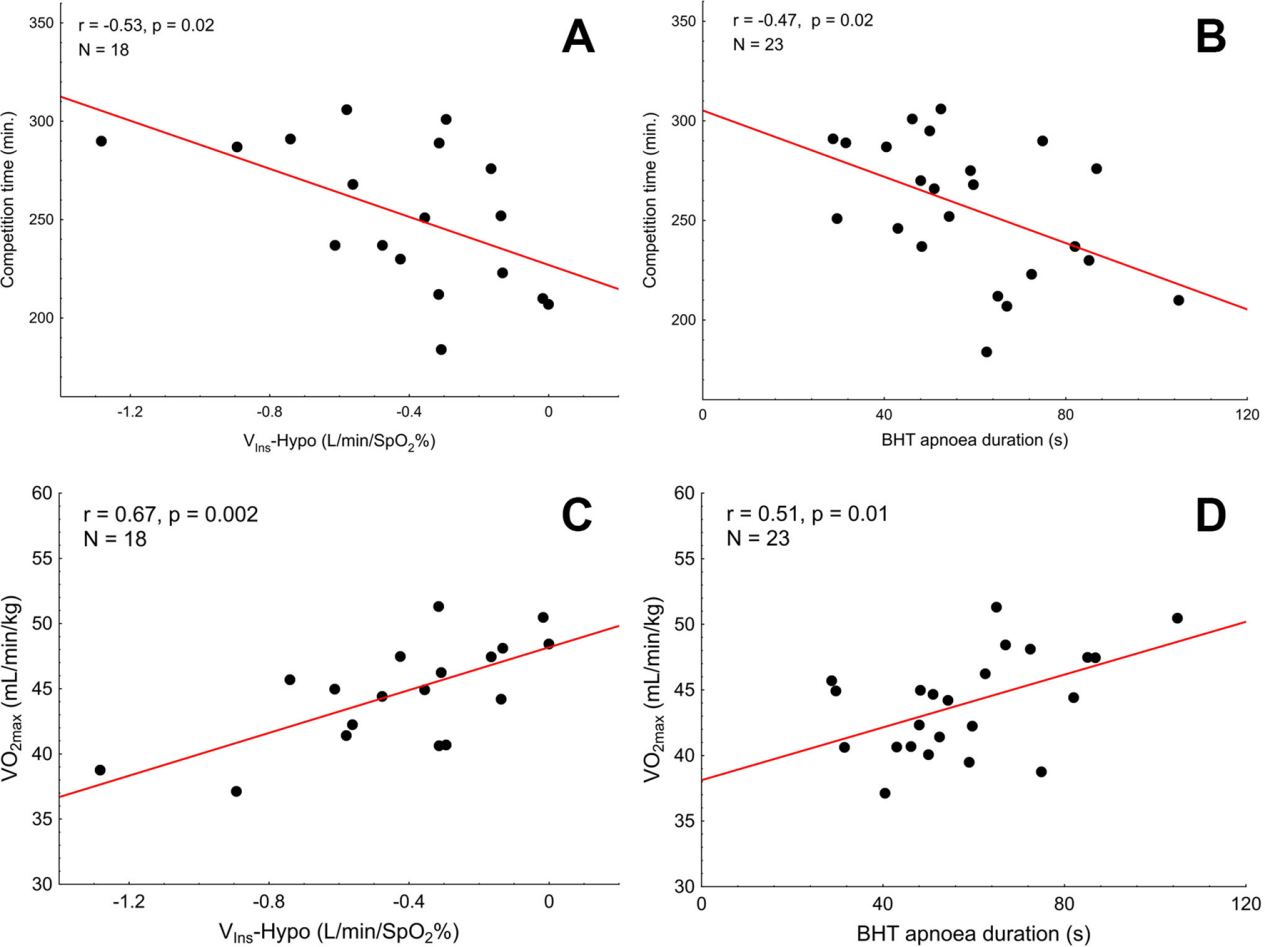
Correlations between the ventilatory response to the chemoreceptor-stimulating manoeuvres (transient hypoxia and breath-holding) and the parameters of individual performance: the marathon time and VO_2max_. (**A**) The competition time plotted against V_Ins_-Hypo (minute ventilation-response to the transient hypoxia). (**B**) The competition time plotted against the pre-race BHT apnoea duration. (**C**) VO_2max_ plotted against V_Ins_-Hypo (minute ventilation-response to the transient hypoxia). (**D**) VO_2max_ plotted against the pre-race BHT apnoea duration.

Marathon time was inversely correlated with a ventilatory response from the peripheral chemoreceptors, as assessed by the transient hypoxia (V_Ins_-Hypo vs. competition time: *r* =  − 0.53, *p* = 0.02, Fig. 4A, V_T_-Hypo vs. competition time, *r* =  − 0.50, *p* = 0.03) and the BHT (pre-race BHT apnoea time vs. competition time, *r* =  − 0.47, *p* = 0.02, Fig. 4B). In line with this, VO_2max_ was correlated with V_Ins_-Hypo (r = 0.67, *p* = 0.002, Fig. 4C), V_T_-Hypo (r = 0.55, *p* = 0.02) and pre-race BHT apnoea time (r = 0.51, *p* = 0.01, Fig. 4D), thereby providing further support for the postulated link between peripheral chemosensitivity and individual performance.

**Figure 5:**
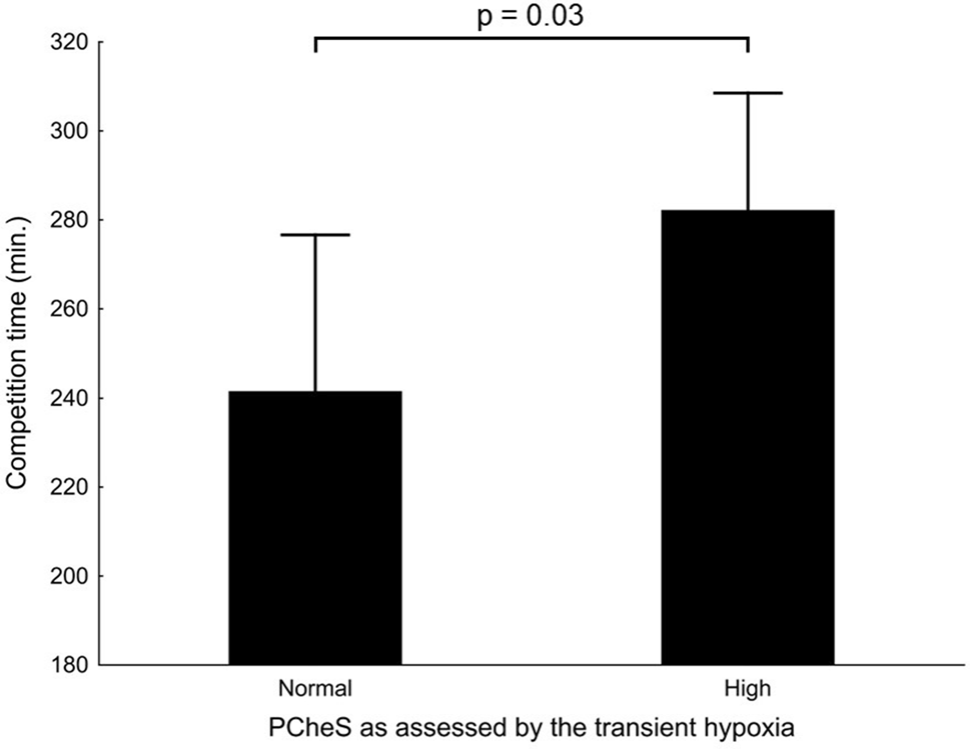
The competition time in subjects with normal versus high peripheral chemoreflex sensitivity as assessed with the transient hypoxia test.

High V_Ins_-Hypo group was characterized by significantly worse competition time as compared with normal V_Ins_-Hypo group (282 ± 26 vs. 242 ± 35 min., respectively, *p* = 0.03, Fig. 5).

Regarding the haemodynamic responses to the chemoreceptor-stimulating manoeuvres, pre-race BHT-SVR slope was correlated with the marathon outcome (r =  − 0.59, *p* = 0.005, Fig. 2D). Figure 2C presents mean beat-to-beat SVR response to the breath-holding test in fast vs. slow finishers (classified according to the median marathon time). We did not observe any significant associations between a change in BHT-SVR slope versus the competition time (r = 0.05, *p* = 0.84) and pre-race BHT-SVR slope or pre- versus post-race change in BHT-SVR slope and VO_2max_ (r = 0.36, *p* = 0.11 and r =  − 0.33, *p* = 0.15, respectively). Similarly, no statistically significant linear correlations were found between the competition time or VO_2max_ and the transient hypoxia test-derived responses: HR-Hypo (r = 0.25, *p* = 0.32 and r = 0.23, *p* = 0.35, respectively), SBP-Hypo (r =  − 0.03, *p* = 0.90 and r = 0.05, *p* = 0.83, respectively), MAP-Hypo (r =  − 0.04, *p* = 0.87 and r = 0.13, *p* = 0.60, respectively), SVR-Hypo (r =  − 0.17, *p* = 0.49 and r =  − 0.30, *p* = 0.23, respectively). Furthermore, we did not find any significant pre-race × change interaction for the BHT-apnoea time (*p* = 0.16 and 0.76, for the marathon time and VO_2max_, respectively) or the BHT-SVR slope (*p* = 0.27 and 0.49, for the marathon time and VO_2max_, respectively).

### Heart rate variability and blood pressure variability

The post-race pNN50, HF-HRV and SDNN were diminished as compared with the pre-race values, while the LF-HRV increased after the race. BPV-SD did not differ between and after the race, whereas LF-BPV was increased after the race (Table 4).

No significant correlations between the parameters of individual performance and pre-race HRV parameters or pre- versus post-race changes in HRV parameters were found, although an inverse correlation between pNN50 and the marathon time approached significance (r =  − 0.37, *p* = 0.08, Table 5). Similarly, we did not identify any significant pre-race × change interactions for HRV indices (Table 5).

**Table 5:** Correlations between the parameters of individual performance (the competition time and VO_2max_) and the HRV and BPV parameters (pre-race values and pre-vs. post-race changes).

	Competition time			VO_2max_		
	Pre-race	% Change	Pre-race × change interaction	Pre-race	% Change	Pre-race × change interaction
SDNN	− 0.27 *p* = 0.20	0.21 *p* = 0.34	*p* = 0.29	0.04 *p* = 0.84	− 0.26 *p* = 0.22	*p* = 0.88
pNN50	− 0.37 *p* = 0.08	0.13 *p* = 0.55	*p* = 0.30	0.17 *p* = 0.45	− 0.22 *p* = 0.31	*p* = 0.53
LF-HRV	0.32 *p* = 0.14	− 0.29 *p* = 0.18	*p* = 0.07	− 0.19 *p* = 0.40	0.25 *p* = 0.24	*p* = 0.18
HF-HRV	− 0.32 *p* = 0.14	− 0.03 *p* = 0.89	*p* = 0.07	0.19 *p* = 0.40	− 0.08 *p* = 0.72	*p* = 0.18
BPV-SD	− 0.42 *p* = 0.04	–	*p* = 0.68	0.64 *p* = 0.001	–	*p* = 0.99
LF-BPV	0.05 *p* = 0.83	0.29 *p* = 0.18	*p* = 0.05	− 0.02 *p* = 0.92	− 0.07 *p* = 0.75	*p* = 0.18

*r* Pearson’s linear correlation coefficients and *p* values are presented for correlations with pre-race values and % change; *p* values for general linear model are presented for pre-race × change interaction.

SDNN, standard deviation of RRIs; pNN50, the percentage of adjacent RRIs with a difference of duration > 50 ms; LF-HRV, the power of HRV spectrum within low-frequency range; HF-HRV, the power of HRV spectrum within high-frequency range; BPV-SD, standard deviation of beat-to-beat SBP; LF-BPV, the power of DBP variability spectrum within low-frequency range.

**Figure 6:**
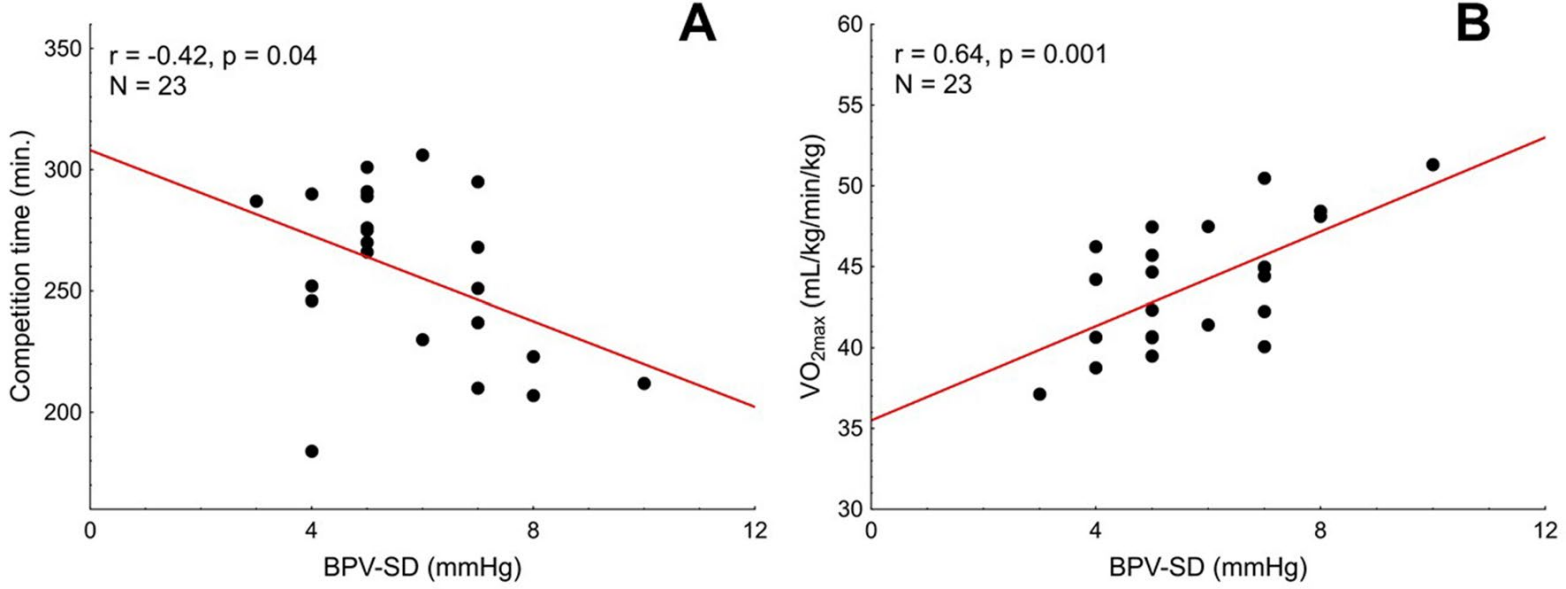
VO_2max_ (**A**) and the competition time (**B**) plotted against the pre-race standard deviation of the beat-to-beat systolic blood pressure (BPV-SD).

**Figure 7:**
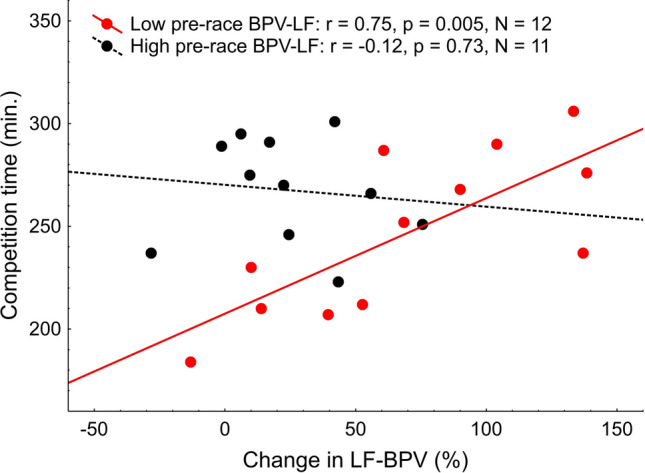
The competition time plotted against the pre- versus post-race change in LF-BPV in the subjects with low (in red) and high (in black) pre-race LF-BPV.

Pre-race BPV-SD was related with both, the competition time (r =  − 0.42, *p* = 0.04) and the VO_2max_ (r = 0.64, *p* = 0.001, Table 5, Fig. 6). We found no correlation between the competition time or VO_2max_ and LF-BPV (pre-race values or pre- versus post-run changes, Table 5), however statistically significant pre-race × change interaction was identified (*p* = 0.05, Table 5). To explore this interaction further, the subjects were divided into low- and high pre-race LF-BPV group according to the median of the pre-race LF-BPV. Interestingly, in the low pre-race LF-BPV group, the competition time correlated strongly with the change in BPV-LF (r = 0.75, *p* = 0.005), indicating that the fast marathon performance is accompanied by little or no increase in LF-BPV in subjects with initially low LF-BPV, whereas no such relation was found in subjects with high LF-BPV before the run (Fig. 7).

### Plasma renin and aldosterone levels

Plasma renin and aldosterone values were elevated after the run (Table 4).

We found no relations between the parameters of individual performance (competition time and VO_2max_) and the pre-race plasma renin (r = 0.11, *p* = 0.61 and r =  − 0.12, *p* = 0.57, respectively) and aldosterone (r =  − 0.20, *p* = 0.37 and r = 0.25, *p* = 0.25, respectively) levels or pre- versus post-race changes in plasma renin (r =  − 0.19, *p* = 0.40 and r =  − 0.03, *p* = 0.90, respectively) or aldosterone levels (r = 0.10, *p* = 0.65 and r =  − 0.19, *p* = 0.39, respectively). There were no significant pre-race × change interactions for renin (*p* = 0.55 and 0.61 for the marathon time and VO_2max_, respectively) or aldosterone (*p* = 0.78 and 0.59, for the marathon time and VO_2max_, respectively).

## Discussion

To our knowledge, this is the first study addressing the utility of the autonomic variables as predictors of the marathon performance in healthy men aged 50 years and older. The pattern of relations we observed is in stark contrast to that reported for younger competitors^8^. We found that: (1) low ventilatory responsiveness to the manoeuvres targeting peripheral chemoreflex (the transient hypoxia test and the breath-holding test), and (2) enhanced vasoconstrictor responsiveness to the breath-holding are the predictors of fast marathon performance in healthy men over 50 years of age; (3) neither the post-exercise vasodilation nor the markers of sympathetic outflow to the vessels (LF-BPV) or sympathovagal control of the heart (HRV analysis) predicts the marathon outcome in this population. The major strength of our study is that we used two different methods for the peripheral chemoreflex sensitivity assessment (the transient hypoxia test and the breath-holding test), as well as two parameters of individual performance, namely the marathon completion time and the VO_2max_ as assessed with the treadmill cardiopulmonary exercise test. Of importance, our major results displayed similar pattern regardless the method for the peripheral chemoreflex sensitivity evaluation considered.

We are not aware of any previous studies demonstrating the relation between peripheral chemoreceptor sensitivity and marathon performance in the middle-aged and older men. Fast performance was associated with low ventilatory response to transient hypoxia and breath-holding in our study. Given that the link between the marathon performance and autonomic regulation of the cardiovascular system (as assessed by HRV and BPV^5,8,14^ and beat-to-beat cBRS^5,8,14^) has been repeatedly reported in younger subjects^1^, the sparseness of data on peripheral chemosensitivity in this context may appear surprising. Of note, however, role of peripheral chemoreflex in regulation of circulation during the exercise has been largely underestimated^37^ until the series of elegant studies by Stickland et al.^15–17^. They have shown, in chronically instrumented dogs, that transient inhibition of carotid chemoreceptors during mild exercise leads to an abrupt vasodilation in the hindlimb, and this effect is abolished by α-adrenergic receptor blockade or carotid body denervation^15^. Subsequently, they translated these findings to humans and found that transient suppression of carotid chemosensory activity during exercise decreases sympathetic vasoconstrictor outflow^16^ and increases muscle blood flow and conductance^17^.

Therefore, given that the peripheral chemoreflex was shown to restrain the exercise-induced vasodilation via increased sympathetic vasoconstrictor outflow^15–17^ and both aforementioned factors—namely, exercise vasodilation and sympathetic regulation of vasomotor tone—were reported to be major predictors of the marathon performance^8^, it is tempting to speculate that the peripheral chemosensitivity influences the marathon outcome by the effect on exercise vasodilation. Alternatively, peripheral chemoreflex may impact the marathon performance by the effect on exercise hyperpnoea. Our results are discussed in relation to both scenarios below, although it needs to be emphasized that the study design does not permit any conclusions regarding causality.

### Does the peripheral chemoreflex impact marathon performance via its effect on exercise-induced vasodilation?

Surprisingly, fast performance was associated with greater SVR increase in response to breath-holding test. Given that an inverse relationship between sympathetic responsiveness and sympathetic tone was reported previously in young volunteers^38^, one might conclude that greater SVR responsiveness to stressors reflects merely a decreased tonic sympathetic outflow to the vessels, resulting in lower SVR at rest. However, resting SVR was similar in fast vs. slow finishers, both before and after the run (Fig. 2C). Clearly, the observation that fast finishers were characterized by low ventilatory and high vascular responsiveness to peripheral chemoreflex stimulation does not support our hypothesis that low peripheral chemosensitivity results in low vasoconstrictor drive to vessels, greater exercise-induced vasodilation and eventually, better marathon performance. Moreover, vascular response to hypoxia was not associated with individual performance in our study.

The following two points should be considered, however. First, chemoreceptors’ ventilatory responsiveness does not necessarily mirror the vascular^27^ or sympathetic^39^ responsiveness and the existence of distinct connections from the carotid body into the nervous system, targeting different reflexes (e.g. ventilatory and sympathetic) was postulated^40^. Second, chemoreceptors’ acute sensitivity to stressors does not necessarily parallel chemoreceptors’ tonicity^40^. In other words, enhanced vascular response to stressor (e.g. breath-holding) is not necessarily a marker of high tonic vasomotor tone (eg. due to increased chemoreceptor tonicity). Sympathetic tone and sympathetic responsiveness should not be equated with one another^38^.

### Does the peripheral chemoreflex impact marathon performance via its effect on exercise hyperpnoea?

Hypoxic ventilatory response at rest was reported to be lower in athletes, including marathon runners, in some^41–44^, but not all studies^45,46^. Mean hypoxic ventilatory response value (defined as change in minute ventilation) in the current study (V_Ins_-Hypo: − 0.42 ± 0.32 L/min/SpO_2_%) was close to the values reported by our^27,47,48^ and other groups^26,28,49^.

Although circulating levels of the major carotid chemoreceptor stimuli (PO_2_, PCO_2_, lactate, K^+^) do not change significantly during the exercise^50^, several lines of evidence support a role of carotid chemoreceptors as a source of tonic excitatory drive for breathing which is equally important at rest as well as during the exercise^50,51^. Firstly, in animal models, carotid body denervation leads to hypoventilation at rest and during exercise^52–54^. Secondly, suppression of peripheral chemoreceptor activity with hyperoxia decreases ventilation at rest and during exercise in humans^55–57^. Thirdly, asthmatic patients who had undergone carotid-body resection for the relief of dyspnoea were characterized by lower rate of increase in ventilation to its steady-state, higher PCO_2_ level and less marked hyperpnoea above the anaerobic threshold as compared with age-matched controls^58^. It should be underlined, however, that there is still huge controversy over whether peripheral chemoreceptor mechanism contributes significantly to the control of breathing during exercise^50,59^.

We did not collect respiratory data during or immediately after the run and therefore the existence of a relation between chemosensitivity and exercise hyperpnoea cannot be verified directly in the current study. However, lack of linear relation between hypoxic ventilatory response and maximal minute ventilation during the cardiopulmonary exercise test (r = 0.03, *p* = 0.89) argues against this explanation.

### Post-competition vasodilation does not predict the marathon performance in the older men

Gratze et al.^8^ found robust correlations between the marathon time and both: (1) the pre- versus post-race change in SVR (*r* = 0.29) and (2) the pre-race LF-BPV (*r* = 0.48) in the group of relatively young males (mean age: ~ 40 y, range: 26–57 y) and concluded that sympathetic vasomotor control and post-competition vasodilation are critical determinants of the marathon outcome. Employing a similar approach, we failed to replicate this finding in healthy men aged 50 years or older. The change in SVR did not correlate with the marathon outcome or VO_2max_ in our study. Regarding LF-BPV, no significant correlations between performance and the pre-race LF-BPV or the change in LF-BPV were found in the whole group. However, analysis of the effect of interaction between the pre-race LF-BPV and the change in LF-BPV revealed that fast marathon time is accompanied by little or no increase in LF-BPV after the run in the subjects with initially low LF-BPV only. Therefore, it is plausible that that the pre- versuss post-race change in the sympathetic vasomotor outflow has a certain prognostic value for athletic performance in middle-aged and older man with low tonic sympathetic drive to the vessels. Nevertheless, predictive value limited to subjects with low resting sympathetic tone precludes the widespread usage of LF-BPV for predicting the marathon outcome. Interestingly, however, the two other physiological measures predicted the competition time in the whole group in our study. We found that fast finishers displayed *higher* vascular responsiveness to the breath-holding test (r =  − 0.59) and greater overall BPV (expressed as BPV-SD, *r* =  − 0.42).

Peripheral vasodilator capacity declines with aging in healthy humans^60^ and this is particularly manifested during the exercise^61,62^. Multiple factors are involved, however, and, for instance, regular aerobic exercise was shown to restore vasomotor responsiveness in older healthy subjects to levels similar to those in young adults^63^. In line with this, an average change in SVR found in the current study (decrease by ~ 20%) is slightly lower as compared with the value reported by Gratze et al. (decrease by ~ 30–35%).

Factors attributed to age-dependent impairment of vasodilator capacity include endothelium-dependent (e.g. decreased NO bioavailability^60^) and non-endothelium-dependent mechanisms (e.g. increased sympathetic vasoconstrictor outflow^62^). As sympathetic vasomotor tone was shown previously to predict the marathon outcome^8^, age-dependent alterations in sympathetic control will be outlined herein. Aging is associated with an increased overall sympathetic activity at rest^64–69^. However, it does not necessarily translate into higher blood pressure and α_1_-desensitization^70^ and impaired functional sympatholysis^62^ have been proposed as underlying mechanisms. In line with this, Hart et al.^71^ reported an inverse relationship between sympathetic vasoconstrictor activity (as assessed directly using microneurography) and vascular conductance in young men (mean age ~ 27 y), while no such relation was found in a group of older men (mean age ~ 61 y). In other words, it seems that neural control of the vessels is impaired in older men. In young adults, cardiac output is a key compensatory mechanism that maintains blood pressure at an appropriate level when tonic sympathetic outflow to the vessels is low (and therefore, SVR is decreased as well)^71^. Literature data regarding the sympathetic vascular responsiveness to stressors in the older are less consistent. The older subjects displayed blunted MSNA response to head-up tilting^68^ or nitroprusside injection^72^, enhanced MSNA response to phenylephrine injection^72^ or whole-body cooling^73^, and unchanged MSNA responsiveness to mental stress^74^.

Keeping in mind that the sympathetic control of the vessels is impaired in the older men, we suggest the following interpretation of our results, revolving around a trade-off between the peripheral vasodilation (aimed to enhance oxygen supply to the working muscles) and a need to maintain blood pressure within a normal range during the exercise. In younger subjects, high maximal cardiac output compensates for an enhanced peripheral vasodilation so effectively that maximal (or close to maximal) level of the peripheral vasodilation may be triggered. As a result inter-individual differences in maximal peripheral vasodilation translate to better or worse perfusion of the muscles, and eventually, better or worse performance. In older subjects however, maximal cardiac output is decreased^75^, and thereby not capable to effectively offset a fall in SVR. Consequently, maximal peripheral vasodilation is rarely achieved and the body’s ability to quickly reroute the blood from one vascular bed to another is by far more important for the individual performance than the maximal achievable level of vasodilation. Given the age-related deterioration of the vascular control, mentioned above, the vascular responsiveness becomes a crucial predictor of the individual performance in the older subjects.

Importantly, our observation that in subjects with low tonic vasomotor drive, better marathon outcome is accompanied by little increase in the vasomotor drive after the run fits into this explanation given that an inverse relationship between the sympathetic responsiveness and the sympathetic tone has been reported^38^. In these subjects, low sympathetic tone during the run could be compensated by the remarkable increase in cardiac output.

Our results corroborate the findings by Ng et al.^76^ who reported higher sympathetic vasomotor control at rest and during the exposure to laboratory stressors (cold pressor test and isometric handgrip) in healthy older (over 60 y) endurance athletes as compared to age-matched untrained controls. Furthermore, this effect may be limited to older athletes, given that in younger subjects, 4–6 weeks of dynamic training was reported to decrease MSNA response to exercise^77,78^.

In the study by Gratze et al.^79^, a subgroup of marathon runners experiencing pre-syncopal symptoms during active standing test (performed 2 h after the run) was identified. The authors claimed, based on LF-BPV changes, that those runners have exhausted their sympathetic reserve – the ability to further increase sympathetic vasomotor drive. These results underscore the importance of the fully functional sympathetic regulation of blood vessels for the marathon outcome. Given that maximal cardiac output decreases with age^75^, preserved vascular responsiveness allowing for redistributing the blood from non-active areas to the working muscles would be of particular importance in the older athletes.

Of note, however, Gratze et al. reported no differences in pre-race values of autonomic and haemodynamic parameters (at rest or during active standing) between the males experiencing- vs. non-experiencing pre-syncopal symptoms. Moreover, the competition time was virtually identical between both groups. In stark contrast, in the current report, fast marathon performance was accompanied by an elevated pre-race SVR responsiveness to a stressor (breath-holding) in healthy middle-aged and older men.

LF-BPV was not related with the parameters of individual performance in the whole group in our study and it corroborates the aforementioned hypothesis based on the impaired neural control of the vessels in the older males. However, another index of the BPV—standard deviation of the beat-to-beat SBP (BPV-SD) correlated with both, the competition time and VO_2max_. We believe it provides further support for our explanation, given that SD-based index is vulnerable to random, non-rhythmic changes in the variable and does not necessarily reflect rhythmical oscillations that may result from tonic neural influences. In this context, BPV-SD is rather a marker of vascular responsiveness to random environmental changes than a marker of a neutrally-driven tonic control of the vasculature, thereby being closer to BHT-SVR than to LF-BPV.

### Does the heart rate variability analysis identify fast finishers among older male marathon runners?

Multiple studies demonstrated that HRV declines with advancing age^80,81^ and training improves HRV in the older individuals^82,83^. Furthermore, training-induced changes in HRV were shown to predict individual performance^5,83–85^. The effect of ageing on HRV appears to be more complex, however. In a large sample of > 1.700 subjects aged from 40 to 100 years, a linear decrease with age in overall autonomic regulation of the heart function was found to be accompanied by the U-shaped pattern of changes of the vagally-mediated indices (e.g. pNN50) with the nadir at the seventh decade^86^. Lack of any significant change in HRV with healthy ageing has been also reported^87^.

In our study, fast finishers tended to display higher values of a vagally-mediated pNN50 parameter before the race (*p* = 0.08). Although taking into consideration a relatively small sample size, we cannot refute the possibility that HRV parameters are linked to the marathon performance in the older athletes, the observation that other ANS-dependent parameters outperformed the most popular HRV indices seems still worth emphasizing.

### Study limitations

We acknowledge that the design of our study precludes conclusions on causal associations. The other limitation of our study is the small sample size which probably constrained the capacity to detect predictors of the individual performance. Nevertheless, we believe utilizing two different methods for the PCheS evaluation and two different indices of the individual performance substantially decreased the risk of false positive results, thereby providing a reasonable compensation for the small sample size. Furthermore, sample size of < 30 subjects is common in the studies of similar-design in this field (e.g. 27 athletes studied before and after Ironman competition in Gratze et al.^14^, 9 athletes studied before and after a mountain marathon in Murrell et al.^88^). The results should be interpreted with caution given the high number of tests performed which increases the probability of false-positive results.

Most previous studies utilized linear regression analysis to identify associations between autonomic variables and athletic performance, despite the fact that these relations do not necessarily follow a linear relationship, as it was shown for cognitive performance^89^. In order to identify possible non-linear relationships, we have used the second-order (quadratic) regression (see: supplementary figure 1). However, the major results of the study did not change.

Furthermore, we did not measure muscle sympathetic nerve activity (MSNA) with microneurography^77,78^. MSNA data would shed more light on the alterations in sympathetic outflow in fast and slow finishers.

Given that the autonomic tonicity cannot be equated to the autonomic responsiveness and both are likely to carry different predictive value, the other standard and well-established stress tests (e.g. active standing test, tilt-table test or the cold pressor test^90^) should be considered in future studies on physiological predictors of athletic performance.

### Conclusions

Low ventilatory response from the peripheral receptors (as assessed by the transient hypoxia or the breath-holding test) is a marker of fast marathon performance and high VO_2max_ in healthy men aged ≥ 50 y. The underlying mechanism remains unknown, given that neither the post-competition vasodilation nor the sympathetic vasomotor tone (as assessed with the LF-BPV) predicted the marathon outcome or VO_2max_ in this population. Interestingly, high vascular responsiveness (as assessed by the SVR change to breath-holding) appears to be beneficial in this population.

## Supplementary Information

Supplementary Information.

## Data Availability

All data relevant to the study are presented in this article. The data are not available due to the potential for a breach of confidentiality.

## Code availability

Commercially available software was used: STATISTICA v.12.1 (Statsoft, Tulsa, OK, USA), LabChart v.8 (Dunedin, New Zealand). Baroreflex sensitivity was calculated using CardioSeries 2.4 available upon request from Dr Daniel Penteado (www.danielpenteado.com).
