# Supplementary material for: Low ventilatory responsiveness to transient hypoxia or breath-holding predicts fast marathon performance in healthy middle-aged and older men
Source: Sci Rep. 2021 May 13;11:10255. doi: 10.1038/s41598-021-89766-4 (PMC8119959; doi:10.1038/s41598-021-89766-4)

Supplementary Figure 1. Pre-race values and pre- vs. post-race percentage differences of the examined haemodynamic and autonomic variables plotted against parameters of individual performance: competition time and VO_2max. Linear regression line along with r and p values (orange) and second-order (quadratic) regression line along with R^2 value (blue) were shown.

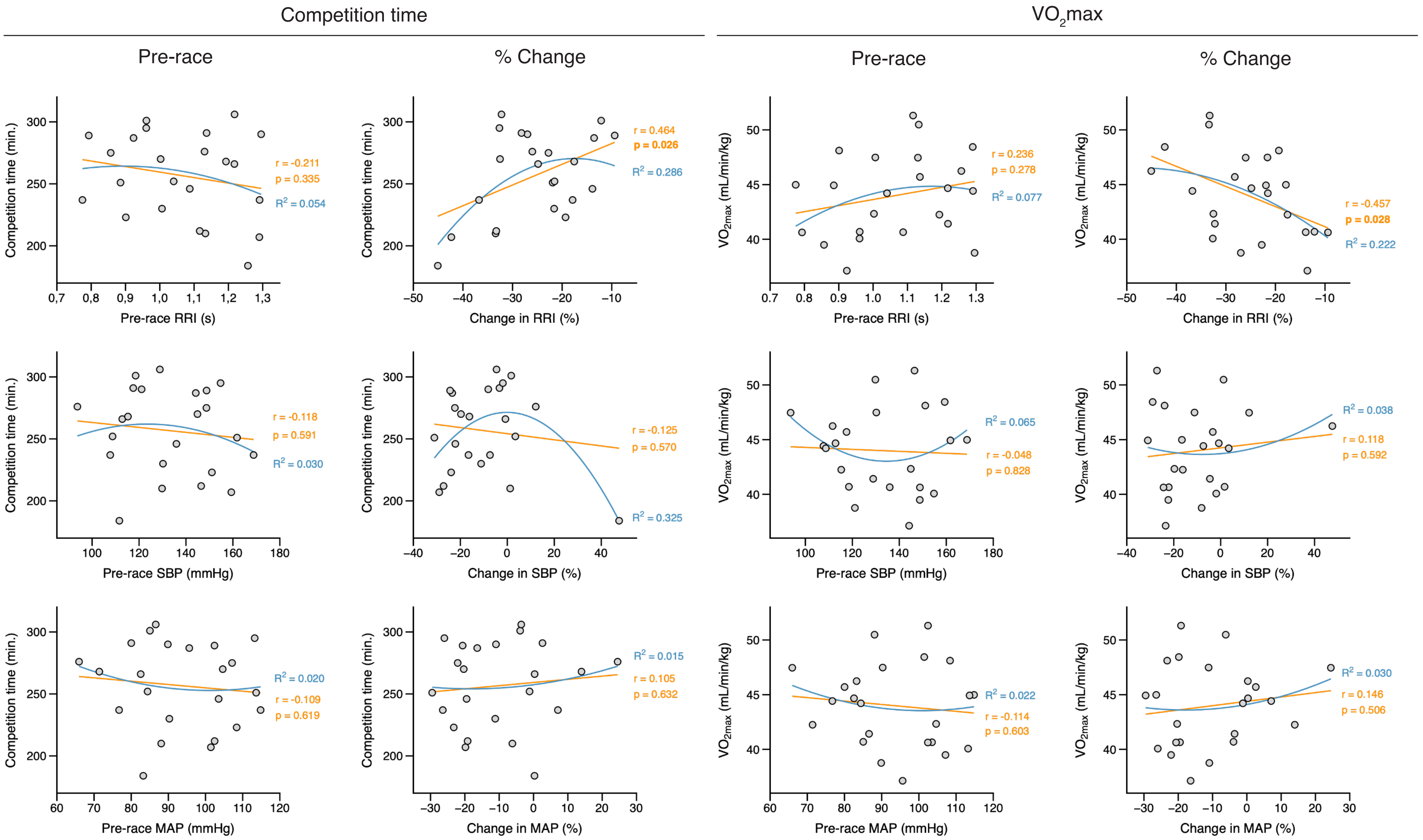

Supplementary Figure 1. (continued)

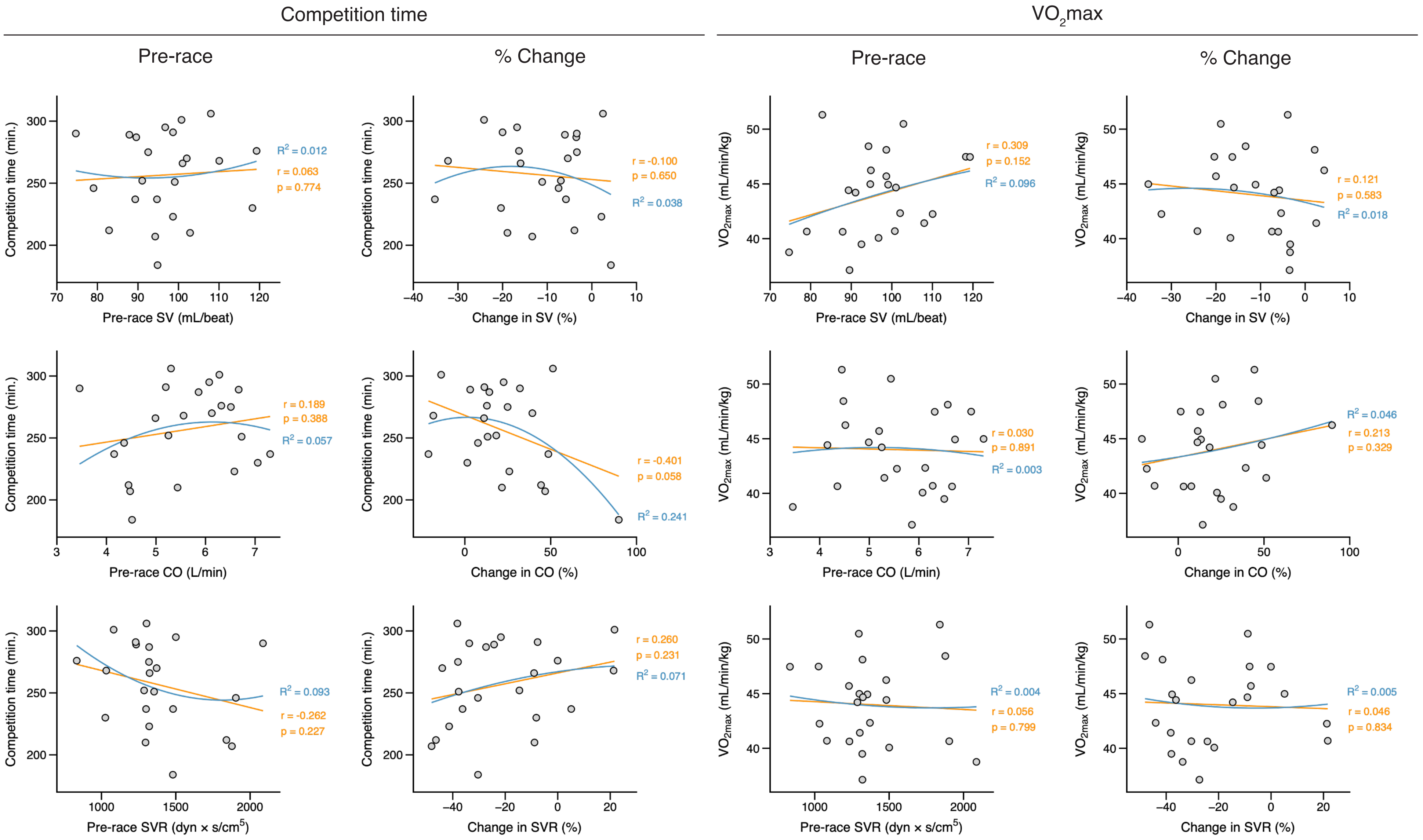

Supplementary Figure 1. (continued)

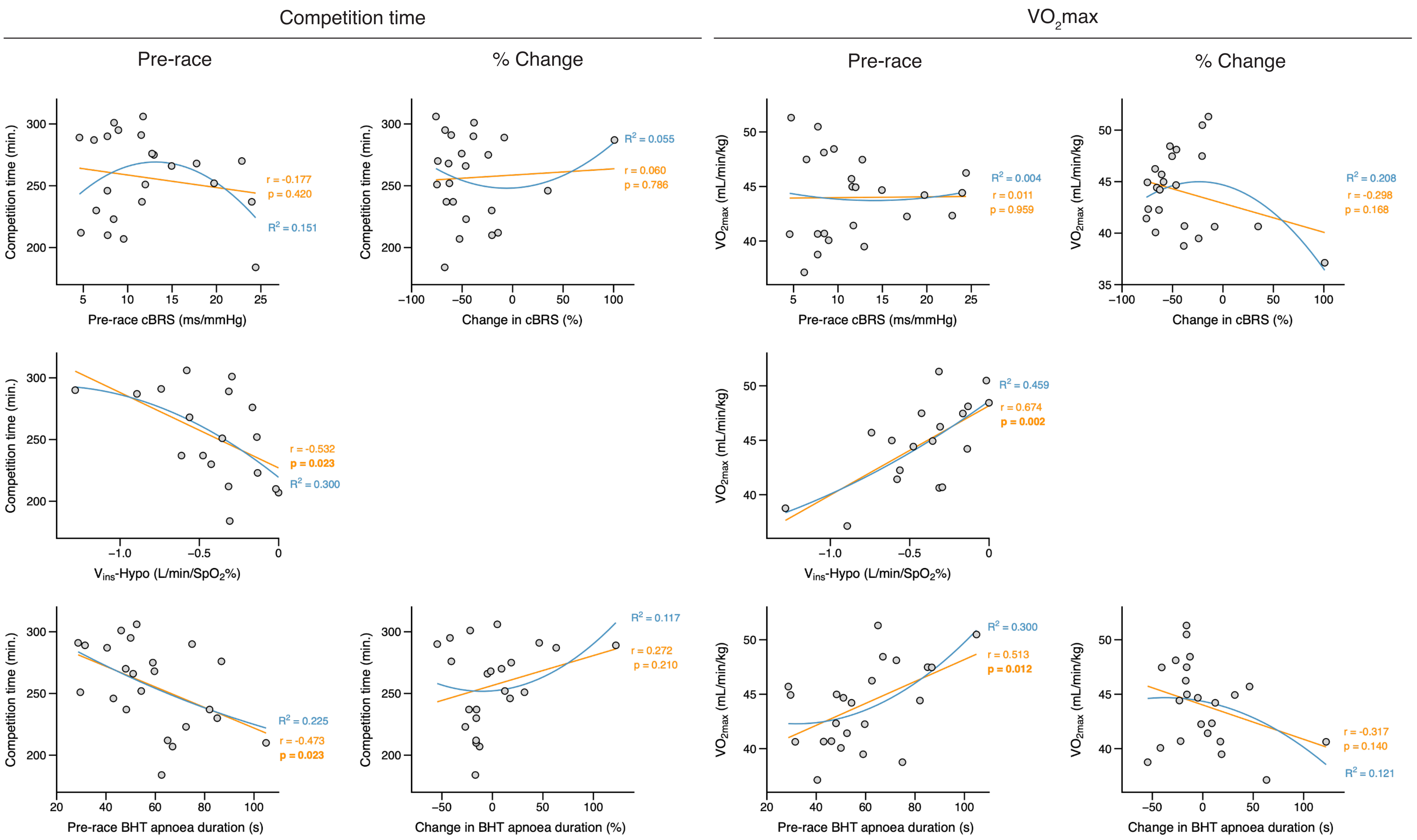

Supplementary Figure 1. (continued)

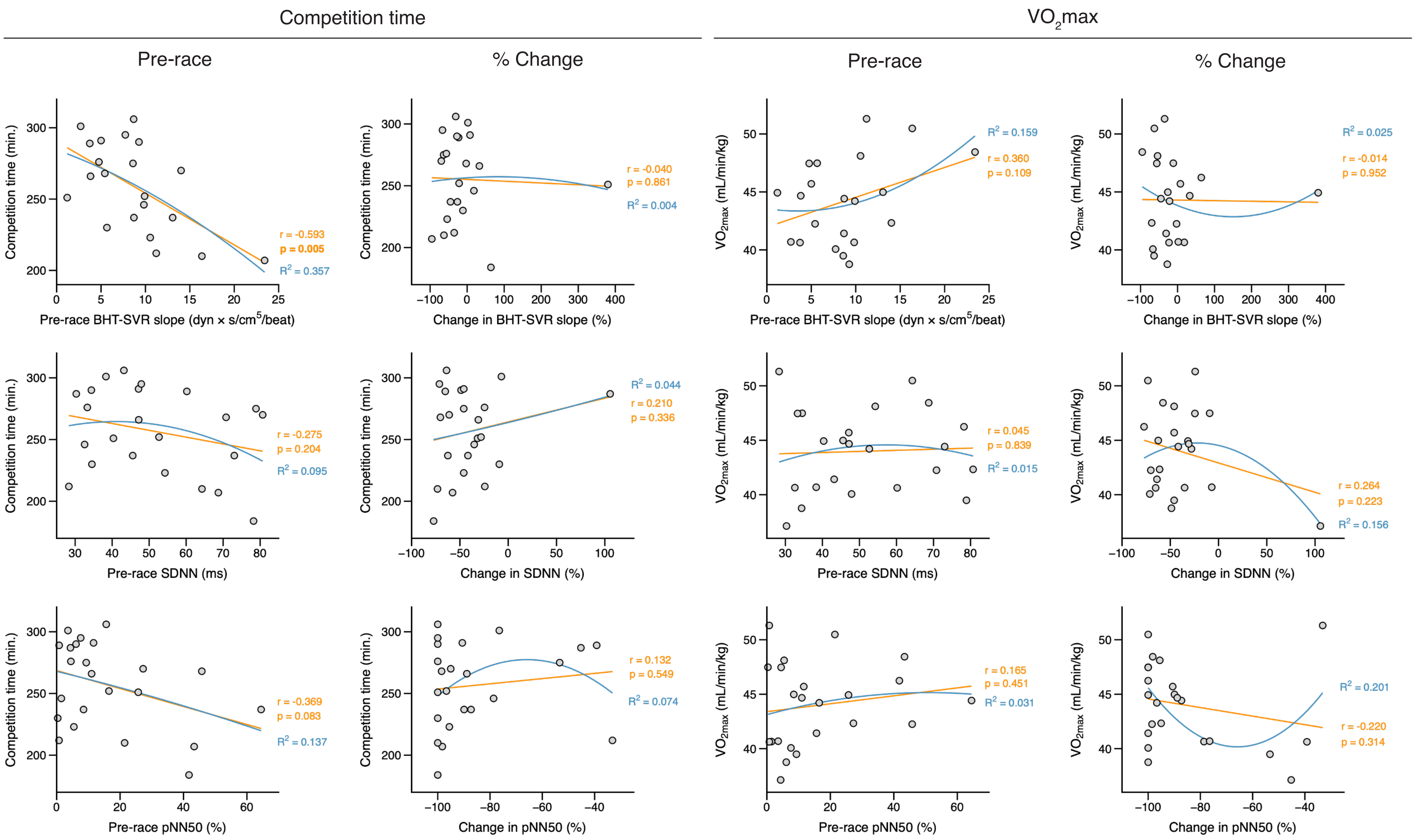

Supplementary Figure 1. (continued)

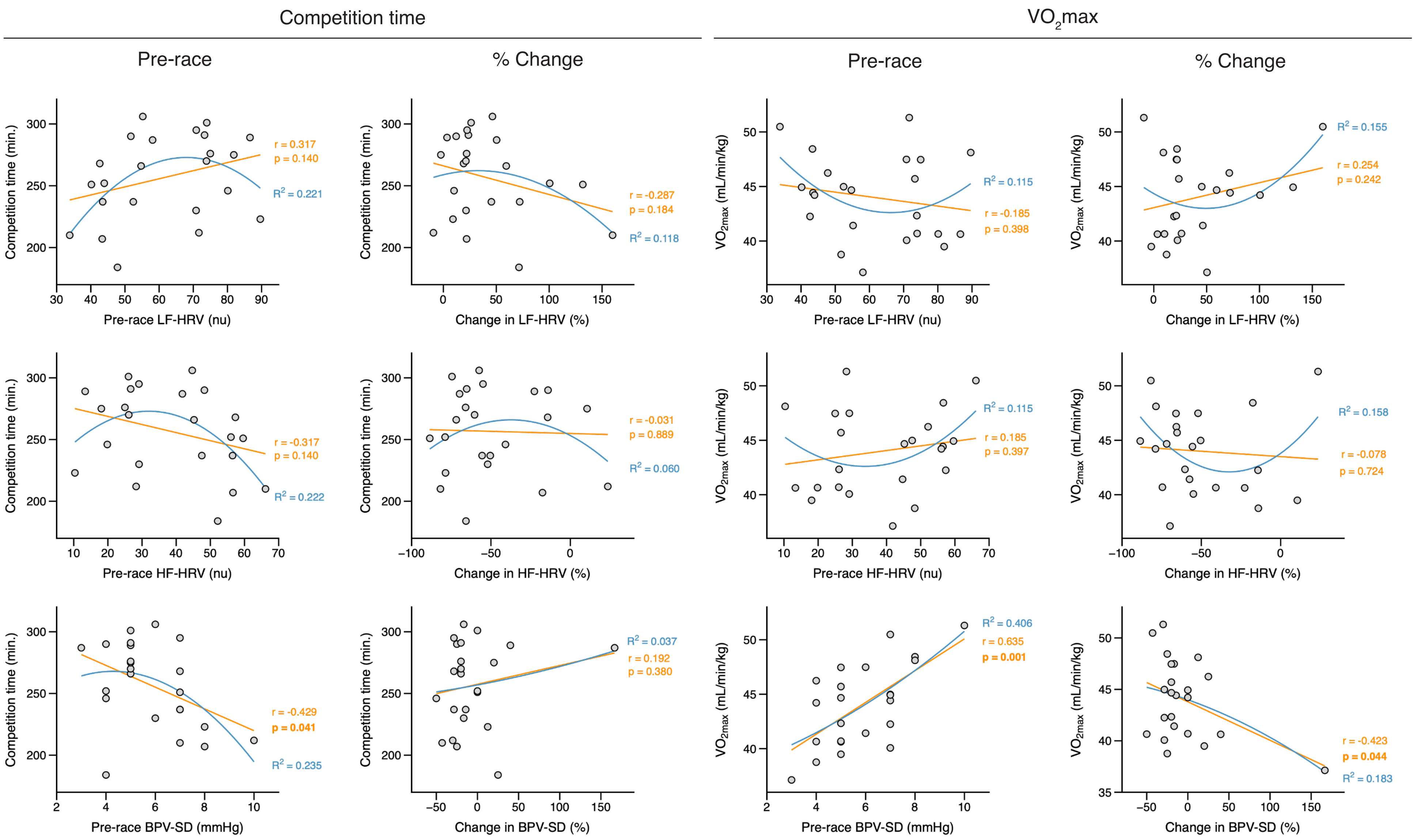

Supplementary Figure 1. (continued)

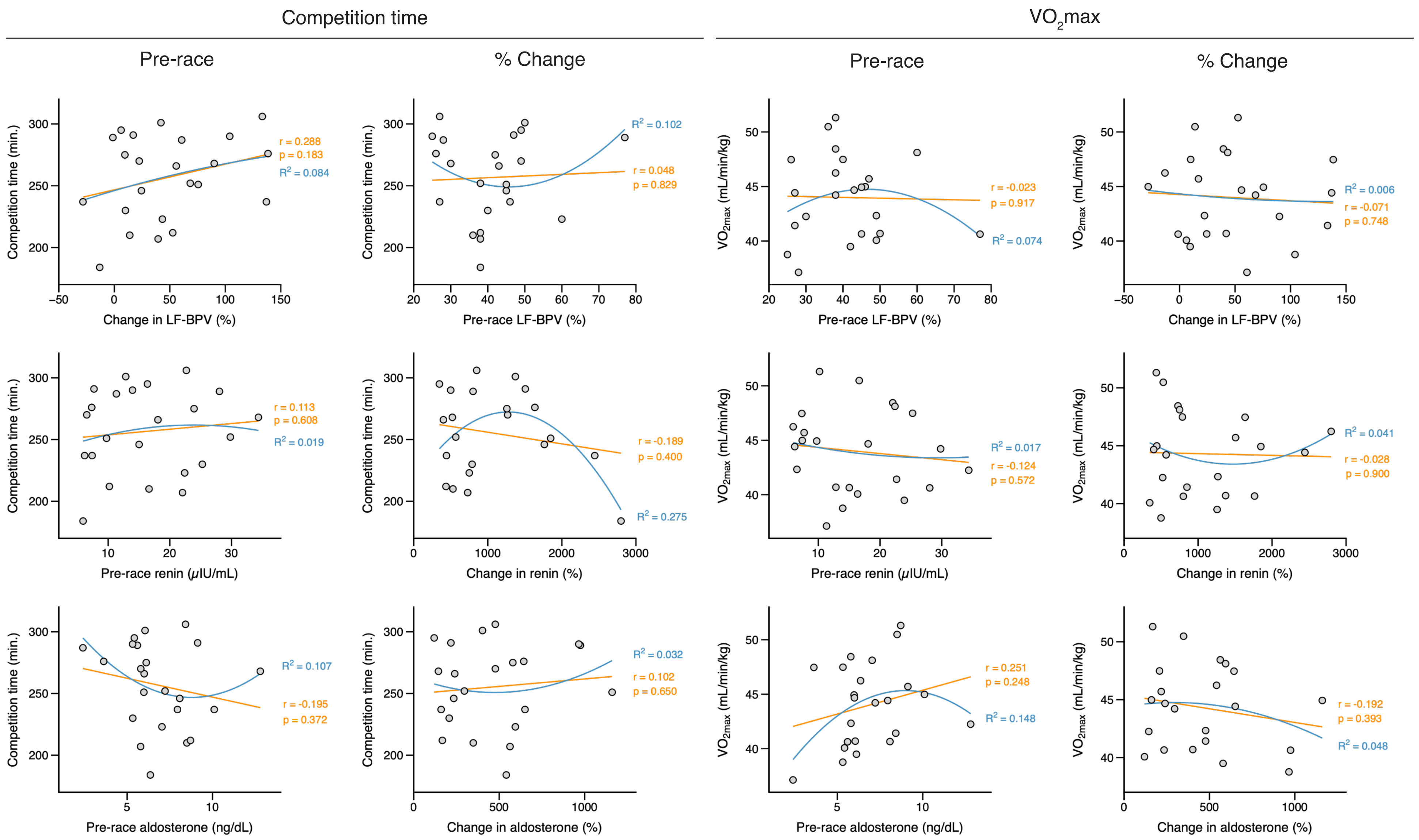

Supplement: Supplementary file 1 — Supplementary Information. [file 41598_2021_89766_MOESM1_ESM.pdf]
